# Provider-initiated HIV testing uptake and socio-economic status among women in a conflict zone in the Central African Republic: a mixed-methods cross-sectional study

**DOI:** 10.1186/s13031-023-00505-0

**Published:** 2023-03-27

**Authors:** Mari Nythun Utheim, Petros Isaakidis, Rafael Van den Bergh, Bantas Bata Ghislain Géraud, Rodrigue Biguioh Mabvouna, Tone Kristin Omsland, Espen Heen, Cecilie Dahl

**Affiliations:** 1 Institute of Health and Society, University of Oslo, Postboks 1130 Blindern, 0318 Oslo, Norway; 2 Médecins Sans Frontières – Southern African Medical Unit, Marshalltown, South Africa; 3 Clinical and Molecular Epidemiology Unit, Department of Hygiene and Epidemiology, University of Ioannina School of Medicine, Ioannina, Greece; 4 Médecins Sans Frontières - Operational Centre Brussels, Operational Research Unit (LuxOR), Brussels, Belgium; 5 Directeur de Ressources, Ministère de La Santé, Bangui, Central African Republic; 6 Department of Biology, University of Roma Tor Vergata, Rome, Italy

**Keywords:** HIV/AIDS, PITC, HIV testing uptake, Family planning, Conflict, Central African Republic, Socio-economic status

## Abstract

**Introduction:**

In the Central African Republic (CAR), HIV/AIDS is the main cause of death in women aged 15–49 years. Increased testing coverage is essential in prevention of HIV/AIDS, especially in areas where conflict hinders access to health care. Socio-economic status (SES) has been shown to be associated with HIV testing uptake. We investigated whether “Provider-initiated HIV testing and counselling” (PITC) could be implemented in a family planning clinic in an active conflict zone in the Central African Republic to reach women of reproductive age and assessed whether socioeconomic status was associated with testing uptake.

**Methods:**

Women aged 15–49 years were recruited from a free family planning clinic run by Médecins Sans Frontières in the capital Bangui. An asset-based measurement tool was created based on analysis of qualitative in-depth interviews. Measures of socioeconomic status were constructed from the tool, also by using factor analysis. Logistic regression was used to quantify the association between SES and HIV testing uptake (yes/no), while controlling for potential confounders: age, marital status, number of children, education level and head of household.

**Results:**

A total of 1419 women were recruited during the study period, where 87.7% consented to HIV testing, and 95.5% consented to contraception use. A total of 11.9% had never been tested for HIV previously. Factors negatively associated with HIV testing uptake were: being married (OR = 0.4, 95% CI 0.3–0.5); living in a household headed by the husband as opposed to by another person (OR = 0.4, 95% CI 0.3–0.6), and lower age (OR = 0.96, 95% CI 0.93–0.99). Higher level of education (OR = 1.0, 95% CI 0.97–1.1) and having more children aged under 15 (OR = 0.92, 95% CI 0.81–1.1) was not associated with testing uptake. In multivariable regression, testing uptake was lower in the higher SES groups, but the differences were not significant (OR = 0.80, 95% CI 0.55–1.18).

**Conclusions:**

The findings show that PITC can be successfully implemented in the patient flow in a family planning clinic, without compromising contraception uptake. Within the PITC framework in a conflict setting, socioeconomic status was not found to be associated with testing uptake in women of reproductive age.

**Supplementary Information:**

The online version contains supplementary material available at 10.1186/s13031-023-00505-0.

## Background

The global epidemic of HIV/AIDS has an unequal geographic effect, and resources are unevenly distributed in the response to the epidemic. Compared to the rest of the world, West- and Central Africa is underserved [1], especially the most unstable countries such as the Central African Republic (CAR) [2] where HIV/AIDS is the main cause of death in people aged 15–49 years [3]. Provision of health care in conflict settings is challenging, and HIV care is no exception. The lack of adapted care for conflict-affected populations with HIV has been pointed out [4, 5], and successful attempts have been made to ensure continued antiretroviral therapy (ART) for patients in conflict settings by adapting the delivery to local circumstances [6].

Further, women and girls are disproportionately affected by HIV/AIDS [7]. This is the case also in CAR—women are hardest hit with 33.7% of deaths in the age group 15–49 estimated to be caused by the disease in 2019 [8]. For men in the same age group, HIV caused 14.2% of deaths (ibid.). In the capital Bangui, HIV prevalence is 10.6% among women aged 15–49 [9]. ART is available, but drug supply and costs can be unpredictable because of insecurity [6], looting and mismanagement of funds [10, 11]. The long and seemingly continuing history of war, violence and insecurity has impeded both provision of health services and research on health.

Increasing testing coverage is one of the key prevention strategies to curb the epidemic [12–15]. Globally, HIV testing coverage is 75%, but this number occludes large geographical disparities [14]. In CAR, HIV testing coverage for the sexually active population was at 3.8% in 2011, which was low compared to other countries [16, 17]. In contexts of generalised epidemics, WHO recommends “Provider-initiated HIV testing and counselling” (PITC), where patients who are already in health facilities for other reasons will be offered an HIV test that they can opt out of. Particular demographics can be reached with early diagnosis and entry to care by introducing PITC in health care settings that are known to and used by that demographic. In the current study, we attempted to evaluate whether PITC in a family planning clinic was successfully reaching women of reproductive age. This is a strategy to seize missed opportunities and increase testing uptake specifically in the vulnerable group of women of reproductive age [18].

Increasing HIV testing uptake is contingent on understanding the barriers to testing. Socio-economic status (SES) could be one such barrier. Studies in Sub-Saharan Africa have shown HIV Voluntary Counseling and Testing (VCT) uptake among women to be positively associated with higher SES, despite free of charge testing [17, 19–21]. PITC has been shown to increase testing uptake in both low, medium and high-income *countries* [22, 23], but the association between PITC uptake and SES of the *patient* has not yet been investigated.

As of today, there is no research on HIV testing uptake and SES in CAR. There are different ways of measuring SES depending on the context [24, 25]. For low-income countries, asset-based measures are common. The assets included vary from one context to another, and can change over time. At the time of this study, the last Multiple Indicator Cluster Survey (MICS) in CAR, measuring SES through household assets and utility services, dated from 2010 [9]. No measurement tool had been developed for post-war CAR. This study had two main aims: to determine uptake of PITC among women of reproductive age in a conflict setting, when the test is integrated in a family planning service, and to investigate whether HIV testing uptake is associated with SES when the test is free of charge. It also seeks to determine whether PITC affects the uptake of family planning. As there is no standard measure of SES in CAR, we needed to complete two additional aims: to develop an asset-based measuring tool to define SES in the target population, and to identify a low time- and resource consuming measure for SES that could be used in later studies.

## Methods

### Study design

This sequential mixed-methods study consisted of two parts: first, qualitative in-depth interviews as well as pilot questionnaire interviews were carried out to develop a measurement tool for SES with context-specific variables. Secondly, a cross-sectional study assessed the uptake of HIV testing and contraception, and examined the association between SES and HIV testing uptake.

### Study setting

**Fig. 1:**
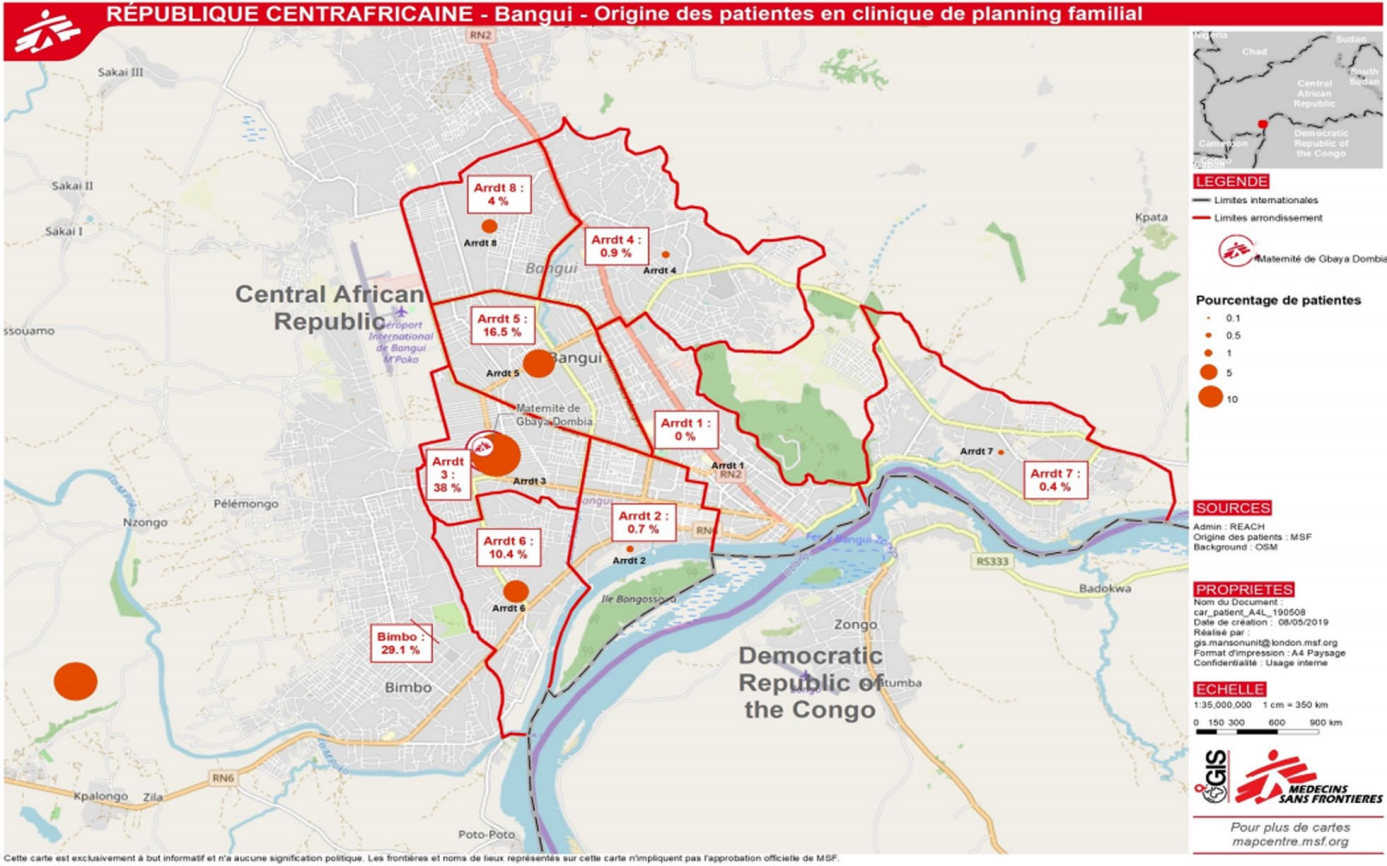
Map of Bangui. Location of Gbaya Dombia maternity, and geographic origin of study participants (N = 1419)

The study was conducted in a family planning clinic within a maternity run by Médecins Sans Frontières (MSF), in the Gbaya Dombia neighbourhood in Bangui. Gbaya Dombia is a densely populated area, close to the main market of Bangui. The neighbourhood is in the part of town that is worst affected by the ongoing armed conflict in CAR. Fires, lootings, and shootings happen regularly in the vicinity, which is also a stronghold for certain armed groups. The maternity provides family planning, HIV-testing, delivery, antenatal care, postnatal care, and emergency services for victims of sexual violence. Due to acts of violence in the vicinity, the clinic also regularly receives victims with stabbing or gunshot wounds for emergency care and referral, although this is not part of the official service package. As all services are free of charge and it is the only MSF family planning clinic in Bangui, clients come from all over the city to receive family planning methods (Fig. 1).

The family planning methods offered were contraceptive pills, injections, implants and condoms. Contraceptive injection was the most popular method. The consultation capacity of the family planning clinic is about 135 clients per week, but client volume varies because of security incidents, making travels unsafe and preventing clients from reaching the clinic. On insecure days, the clinic would receive none or perhaps a handful of clients from the immediate neighbourhood. On secure days, the clinic would often receive its maximum capacity of clients. Until 2017, free PITC and referral to ART was only offered to victims of sexual violence and women in the delivery unit. In 2017, MSF also implemented it as a part of the family planning package. The patient flow was re-organized in order to include the HIV testing while the client was waiting for another consultation, so she would not be required to stay longer or, as for all the services in the clinic, to pay anything (Annex 1).

### Study population, sample and recruitment

#### Qualitative interviews

Women representing different levels of SES were sampled using purposive selection based on two factors—first, a team of local community health promoters advised on the selection, secondly interviewees were asked to self-identify as poorer, average or richer than their peers. Inclusion criteria were that they lived in Bangui and were aged between 15 and 49 years. For the pilot questionnaires, a larger group of women were selected using the same criteria in addition to income source and amount.

#### Quantitative study

All women of reproductive age (15–49 years) living in or around Bangui and attending the family planning service between October 2018 and January 2019 were invited to participate. Minors (15–17 years) were included only with consent of a parent in addition to their own consent. Pregnancy tests were offered to clients during the family planning consultation, and pregnant women were excluded from study participation due to a different medical protocol for ART in case of a pregnancy, as well as no longer being eligible for family planning.

#### Patient flow

The family planning clinic functioned on a first come-first served basis, which meant that a long queue formed in the early morning and patients waited for some time before their consultation. General information about the contraception and HIV service, as well as information about the current study, was presented by the staff in the waiting area. As part of the patient flow, all patients came into a private room where they received an individual explanation of the study and could confidentially choose whether to consent to participation. This happened after their initial PITC consultation, while they were waiting for the test result. They then continued to the family planning consultation (Annex 1).

#### Sample size

For qualitative interviews, sample size was determined by saturation (see data collection). For pilot questionnaires, inclusion went on purposively until there was enough variation in the responses to distinguish three different groups—a total of 20 pilot questionnaires were administered. For the main data collection, sample size was determined based on studies from Zambia [19], Ethiopia [20] and Nigeria [21], allowing us to assume testing uptake of approximately 90% in the mid and high SES groups and 85% in the low SES group. Sample size requirements were calculated as n = (Zα/2 + Zβ)2*(p1(1 – p1) + p2(1 − p2))/(p1 − p2)2, where p1 is the proportion of group 1 (low SES), p2 of group 2 (mid-high SES), α is the probability of type I error, β is the probability of type II error, and Z is the value of α or β. Alpha probability was set to 0.05 and power to 80%. This indicated a need of 563 participants in the low SES group and 844 in the mid and high SES groups: a total of 1407 participants were needed for the study. 1510 clients were seen by the research assistant and were offered study participation. Of these, 59 clients under 18 were excluded from participation, having no parent present for consent, and three patients were excluded because of pregnancy. 29 clients declined to participate. 1419 eligible clients consented to participate in the study. Inclusion rate was thus 94% and participation rate 98%.

### Data collection

As a preliminary study to inform the choice of variables to include in a SES questionnaire, in-depth interviews were carried out with six women in the target population. A semi-structured interview guide was used. Five participants were interviewed in the local language, Sango, using an interpreter. The last one preferred French. Interviews were noted, transcribed into French and analysed thematically to identify factors that can distinguish SES levels in the local context.

Based on the information collected, variables and a points matrix to distinguish levels of each variable were included in a preliminary questionnaire, which was pilot tested. Questions and wording were adjusted to be locally culturally pertinent and understandable, and expanded to capture distinguishable variation between three levels of SES. The finalised questionnaire was administered to the recruited participants, who received an anonymous participation number. Questionnaire data for all 1419 participants were collected by one trained female research assistant. Data collection took place from October 2018 to January 2019.

### Variables

#### HIV testing and contraception uptake

PITC uptake data (yes/no) were recorded by the HIV counsellor who performed the HIV consultations, then reported back to the research assistant at the end of each day. Contraception uptake (yes/no) was recorded in patient medical files by the midwife as per standard routines in the consultation both for new and returning patients, then collected from the file by the research assistant at the end of each day and entered in the participant form. Participant participation numbers were matched to names in a register. The research assistant used this register to retrieve the correct medical files.

Age in years; area of residence; previously tested for HIV (never tested or year of last test); knowledge of status (known/unknown) were also collected.

#### Questionnaire data (Annex 2 and 3)

Information on 16 variables was collected from the questionnaire, as detailed in Table 1: level of education (both in number of years and level obtained); marital status; type of housing; time since last move; number of children < 15 years; number of children < 15 years to feed; % household members < 15 years; head of household; source of income; second source of income; number of household members per breadwinner; savings in Central African Francs (FCFA) per week; mobile phone use as amount spent on credit per week; access to phone in household (yes/no); number of days per week with enough food and last food shortage.

**Table 1:** Point matrix for SES measures

Variable	0 points	1 point	2 points	3 points	4 points	5 points
N children to feed	> 3	3	2	1	0	
N people per breadwinner	> 9	5.8–9	2.4–5.7	1–2.3		
% of household < 15 years	> 60	50.1–60	44.5–50	33.4–44.4	0–33.3	
Saving per week	None	< 1000	1000–2900	3000–5900	> 5900	
Phone use	No phone	0	1–499	500–999	1000–1499	Over 1500
Income type 1	Nothing	Agriculture	Family member	Trade/services	Paid work	
Income type 2	Nothing	Agriculture	Family member	Trade/services	Paid work	
Housing type	Homeless	Camp	Host family	Rented house	Owned house	
Last food shortage	Yesterday	This week	Last week	Last month	Never	
Enough food (days per week)	0	1–3	4–6	7		
Last move	< 2 months	2–6 months	7–12 months	13 months- 5 years	6–10 years	> 10 years
Phone in household	No	Yes				
N children < 5 years*	Over 3	3	2	1	0	
Head of household*	Participant	Acquaintance/ relative	Grandparents	Parents	Partner	Husband
Marital status*	Widowed	Divorced	Single	Cohabitating	Married	
Education level*	None	Pre-school	Primary	Middle	High	University

^*^Also a potential confounding variable

#### SES measures

Participants were given a total asset-based SES score by adding up points attributed to each variable in a point matrix in Table 1.

**Fig. 2:**
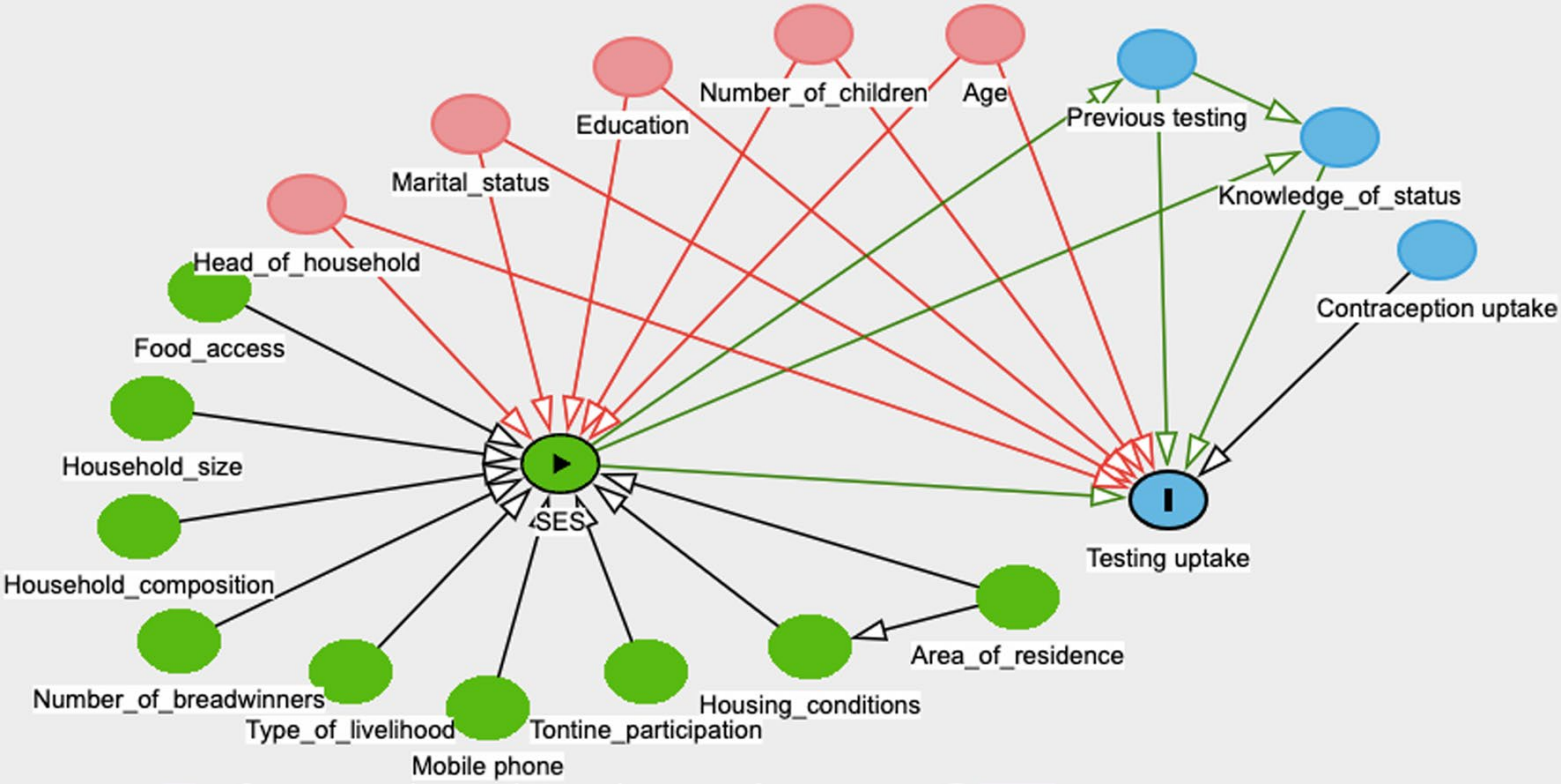
Directed Acyclic Graph (DAG) of hypothesized associations between exposure, outcome and co-factors*. www.dagitty.net. *Green dot with a triangle = Exposure: the composite SES Blue dot with a rectangle = outcome: testing uptake Green dot = Ancestor of exposure: variable affecting the exposure only Red dot = Confounder: variable affecting both exposure and outcome Blue dot = Ancestor of outcome: variable affecting the outcome only

Five SES measures were calculated based on the point matrix, as seen in Table 2. SES 1 included all 16 variables. SES 2 was calculated excluding head of household, marital status, education and N children < 15 years, which were considered to be potential confounding variables in the DAG (Fig. 2) as they can be associated both the exposure SES and the outcome testing uptake. Principal Component Analysis (PCA) was performed and allowed calculation of three further SES measures (SES 3–5) by including only the variables that had a factor score above 0.3 on components that were above the drop on Cattell’s scree test [26]. These three measures are thus composed only of the variables that loaded on the first three PCA components.

**Table 2:** Variables included in SES measures

SES 1 (range: 0–63 points)	SES 2 (range: 0–45 points)	SES 3 (PCA, range 0–21 points)	SES 4 (PCA, range 0–15 points)	SES 5 (PCA, range 0–31 points)
N children to feed	N children to feed	N children to feed	Income 1	N children < 15
% household < 15 years	% household < 15 years	Last food shortage	Income 2	% household < 15 years
People per breadwinner	People per breadwinner	Weekly food shortage	People per breadwinner	Phone use
Housing type	Housing type	Phone in household	Saving per week	Housing type
Last move	Last move	Head of household		Head of household
Income 1	Income 1	Marital status		Marital status
Income 2	Income 2			Education
Phone use	Phone use			
Phone in household	Phone in household			
Saving per week	Saving per week			
Last food shortage	Last food shortage			
Weekly food shortage	Weekly food shortage			
Head of household				
Marital status				
Education				
N children < 15 years

### Statistics

IBM SPSS (version 26) was used for analysis. Cases with any missing data were excluded, leaving measures applicable for 1385 participants. Nominal bivariate analyses were performed with Chi square tests, and Fisher exact tests when numbers were low. Independent samples t-test was used to evaluate differences in mean outcome for continuous variables. Logistic regression (odds ratio (OR), 95% confidence intervals (CI)) was used to investigate the association between each of the five measures of SES (continuous and in 3 groups), and the binary outcome of HIV testing uptake. Based on literature search, hypothesized associations between exposure, outcome and co-factors were assessed using a Directed Acyclic Graph (DAG) in Fig. 2. Potential confounders were included in a multivariable regression model. Two-sided p-values < 0.05 were considered statistically significant.

#### Sensitivity analyses

As food security was found to be an important factor in the qualitative interviews, sensitivity analyses were performed by double-weighting variables on food security. In another sensitivity analysis, participants who had been tested in the last year, i.e. since the beginning of 2018, (N = 988) were excluded, to account for the fact that people may refuse testing if they have been tested recently. Sensitivity analysis on the PCA measures was done by creating an SES measure including the variables that loaded more than 0.7 on each component, and testing for association with HIV testing uptake. Lastly, each confounding variable was checked separately by logistic regression.

## Results

### Participant characteristics

The six interviewees for the qualitative part all lived in the 5^th^ arrondissement, were aged from 21 to 60, and had at least one child. Their education ranged from none to 1^st^ year of university, and they had various sources of income. Background characteristics of participants in the quantitative study are presented in Additional file 1: Table 1. The mean age was 24.3 years (SD 5.04). Most of the women, almost 60%, were aged 18–24 years. Approximately 19% were married, whereas more than half were cohabitating with a partner. The large majority (95%) had children under the age of 15 years, 74% and had between 1 and 3 children, with a median of 2 children. Respondents had a mean of 7.8 years of education, i.e. had started, but not completed, secondary education. 10% of the women had never attended school.

### In-depth interviews

All six interviewees mentioned a stable access to food and strong social support as characteristics of the wealthy. In this setting, these are more reliant factors than material household items. Most interviewees mentioned widespread looting happening at regular intervals. Household items would therefore come and go, and investments made may be lost in the next episode of civil unrest. People with a social network such as family support or business contacts, would most likely recover from the losses quicker than those who were alone. A questionnaire was therefore designed to be sensitive to social support and food supply in addition to material assets.

### Socio-economic characteristics

Socio-economic characteristics of the participants in the quantitative part (N = 1419) are found in Additional file 1: Table 1. The median number of household members was 6, ranging from 1 to 47. More than a third of participants lived in households with more members under the age of 15 than above. Almost two thirds of households had one sole breadwinner. Breadwinners had on average 5.7 people to support, ranging from 1 to 28.

The 29% participating in informal saving groups, “tontines” (a typical local system for collective saving outside of banking systems), saved a mean of 3311 FCFA/5.5 USD (SD 2409.7) per week. Those owning a mobile phone spent a mean of 743 FCFA/1.2 USD per week on credit (SD 733). A substantial 39.5% of participants reported being short on food at least one day every week.

### HIV testing

The measured outcome was HIV testing uptake (yes/no), where 87.7% accepted testing (yes). Those who had not been tested recently (since the beginning of 2018), were significantly more likely to accept testing (OR = 3.8, 95% CI 2.3–6.0). 13.5% of those who were tested had never been tested previously. Contraception uptake was 95.5%, on par with uptake before implementation of HIV PITC which was at 94% the month prior to the beginning of the study. Contraception uptake did not vary significantly according to testing uptake. Married women had a 60% lower odds of opting to get tested (OR = 0.4, 95% CI 0.3–0.5 crude), as did women in households headed by their husband (OR = 0.4, 95% CI 0.3–0.6). Younger women were less likely to get tested (OR = 0.96, 95% CI 0.93–0.99), every increase of 5 years in age meant an 18% higher chance of testing uptake.

**Fig. 3:**
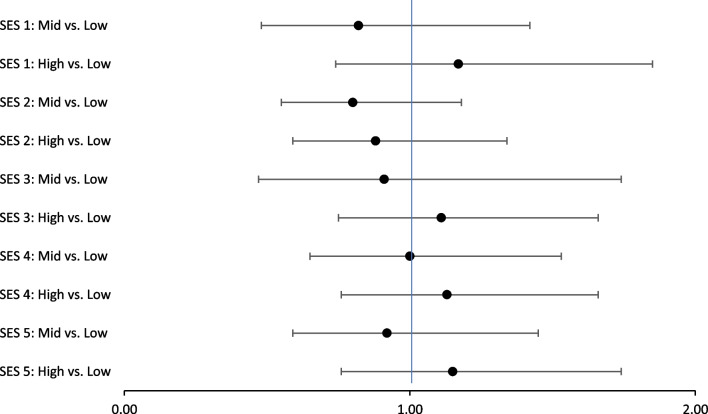
Forest plot displaying odds ratios (95% Confidence intervals) for SES 1–5* with the outcome HIV testing uptake (yes/no), controlling for age, marital status, education and number of children under 15

The matrix-based measures SES 1 and 2 (Table 2) were not statistically significantly associated with testing uptake (Table 3 and Fig. 3). SES 1 was also tested as a continuous variable, but was not significantly associated with testing uptake whether crude (OR = 0.99, 95% CI 0.97–1.02) or adjusted (OR = 1.01, 95% CI 0.97–1.04).

**Table 3:** The association between SES and HIV testing uptake among women aged 15–49 years from a family planning clinic in Bangui, CAR. N = 1385. Odds ratios (95% Confidence intervals) for SES 1–5 with the outcome HIV testing uptake (yes/no)

Exposure	N	Crude	Adjusted									
			Age	Education	Marital status	Head of household	Number of children < 15	Age + education	Age + marital status	Marital status + children < 15	Education + marital status	Age + education + marital status + children < 15
SES 1^a^												
Low	512											
Mid	452	1..09 (.73–1.62)	1.00 (.67–1.45)	1.07 (.72–1.60)	1.19 (.80–1.78)	1.20 (.80–1.79)	0.97 (.65–1.46)	0.99 (.66–1.49)	1.11 (.74–1.67)	1.11 (.73–1.67)	1.25 (.83–1.88)	1.15 (.76–1.74)
High	421	.83 (.57–1.22)	.74 (.50–1.10)	.80 (.53–1.20)	.94 (.64–1.39)	.93 (.63–1.38)	.69 (.46–1.03)	.73 (.48–1.10)	.85 (.57–1.27)	.83 (.54–1.27)	1.04 (.68–1.59)	.92 (.59–1.45)
SES 2^b^												
Low	490											
Mid	519	1.09 (.75–1.59)	1.03 (.70–1.50)	1.08 (.74–1.58)	1.15 (.79–1.68)	1.16 (.79–1.70)	1.01 (.69–1.48)	1.03 (.70–1.51)	1.09 (.74–1.61)	1.10 (.74–1.67)	1.19 (.81–1.75)	1.13 (.76–1.66)
High	376	1.01 (.68–1.52)	.96 (.64–1.44)	1.01 (.67–1.52)	1.00 (.66–1.50)	1.04 (.70–1.57)	.92 (.61–1.39)	.97 (.64–1.46)	.95 (.63–1.44)	.94 (.62–1.42)	1.05 (.69–1.59)	1.00 (.65–1.53)
SES 3^c^												
Low	526											
Mid	679	.96 (.66–1.38)	.88 (.61–1.28)	.97 (.67–1.40)	1.19 (.80–1.75)	1.02 (.69–1.52)	.91 (.63–1.32)	.89 (.61–1.30)	1.09 (.73–1.63)	1.13 (.76–1.67)	1.22 (.83–1.81)	1.11 (.75–1.66)
High	214	.44 (.29-.68)**	.42 (.27-.65)**	.42 (.27-.66)**	1.08 (.57–2.05)	.51 (.29-.90)*	.45 (.29-.69)**	.40 (.25-.62)**	.94 (.49–1.81)	1.01 (.53–1.92)	1.08 (.57–2.04)	.91 (.47–1.74)
SES 4^d^												
Low	674											
Mid	358	.96 (.65–1.43)	1.06 (.71–1.58)	.96 (.65–1.43)	.82 (.55–1.23)	.87 (.58–1.30)	1.02 (.68–1.52)	1.06 (.71–1.58)	.90 (.59–1.36)	.86 (.57–1.29)	.81 (.54–1.21)	.88 (.59–1.34)
High	383	.83 (.57–1.21)	.89 (.61–1.31)	.84 (.57–1.22)	.76 (.52–1.11)	.80 (.55–1.17)	.87 (.60–1.28)	.89 (.61–1.31)	.80 (.55–1.18)	.78 (.53–1.15)	.75 (.51–1.10)	.80 (.55–1.18)
SES 5^e^												
Low	542											
Mid	452	1.28 (.86–1.90)	1.11 (.74–1.67)	1.21 (.80–1.83)	1.29 (.86–1.93)	1.38 (.92–2.06)	.97 (.63–1.50)	1.06 (.69–1.62)	1.16 (.77–1.76)	1.10 (.71–1.70)	1.38 (.91–2.11)	1.17 (.74–1.85)
High	424	.84 (.58–1.22)	.70 (.47–1.03)	.76 (.49–1.17)	.94 (.64–1.37)	.95 (.65–1.39)	.55 (.35-.86)**	.64 (.41-.99)**	.80 (.54–1.20)	.71 (.44–1.15)	1.08 (.69–1.71)	.82 (.48–1.42)

^*^p < 0.05

^**^p < 0.01

^a^Composed by all 16 variables

^b^Composed by 12 variables, confounders are left out

^c^Composed by the 6 variables loading on component 1

^d^Composed by the 4 variables loading on component 2

^e^Composed by the 7 variables loading on component 3

### Principal component as proxy for SES

None of the components in the PCA analysis explained a substantial amount of variation in the sample (maximum 16.1%). The component with highest eigenvalue (SES 3) included the variables marital status, weekly food shortage, last food shortage, number of children to feed and phone in household, with a factor score above 0.3. Among the PCA-based measures, the highest level of SES 3 had a significantly lower testing uptake compared to the lowest level (OR = 0.44, 95% CI 0.29–0.68). The association disappeared when controlling for marital status (OR = 1.08, 95% CI 0.57–2.05). Marital status in itself explained most of the variance in SES 3, and was associated with testing uptake.

### Sensitivity analyses

When the model was restricted to participants “not tested in 2018 or 2019” (N = 423) the negative association between high SES and HIV testing uptake was stronger (OR = 0.47, 95% CI 0.16–1.39) but not statistically significant. The model was also tested with a measure of SES with double weighting of food security variables – association with testing uptake was almost the same and not significant. No significant association was found between the SES measure including the variables that loaded more than 0.7 on each component, and HIV testing uptake (OR = 0.98, 95% CI 0.89–1.08). Marital status and head of household were significantly associated with the outcome, where married women and women living in households headed by their husband were less likely to accept HIV testing. Younger women were significantly more likely to get tested. Education and number of children were not significantly associated with the outcome.

## Discussion

In this study of 1419 women aged 15–49 years attending a family planning clinic in CAR, testing uptake was high, at 87.7%. There was high and unchanged contraception uptake. Although HIV testing uptake was somewhat lower in the high SES groups, we found no significant association between SES and testing uptake, regardless of whether an asset-based or factor-based measure of SES was used. However, marital status was strongly and significantly associated with testing uptake.

In the current study, HIV testing uptake was higher than in comparable studies, although those studies are not from conflict settings. In previous studies on PITC from Zambia, Ethiopia, Zimbabwe and South Africa, uptake ranges from 43.5% to 70.5% in clinic-based samples [27–30]. The South African study investigated testing uptake among women and reported an uptake of only 43.5%, but the most common reason for refusal was to have been recently tested. The difference in uptake might be that testing in general was more available in South Africa than in CAR. Our results may thus be a reflection of the overall health situation in CAR; the country is ranked number 162 out of 162 countries on the Sustainable Development Goals (SDG) index for 2019 [31], with a widespread lack of health services including access to HIV testing.

We found a somewhat lower testing uptake among the higher compared to the low SES group in the current study from CAR. As opposed to our results, studies from Nigeria [21] and Ethiopia [20] showed that HIV testing uptake among women increased incrementally with SES. These studies had community-based sampling in a VCT setup and explain the association as possibly due to opportunity cost of testing. In the PITC setup, we excluded cost and opportunity cost as barriers to HIV testing in the sample, and made the test equally available to all participants. Our study indicates that low SES was not a barrier to HIV testing. Although we cannot exclude opportunity cost entirely as our sample is not community based, there is variation of SES in the sample, which can indicate that the clinic is accessible to women of varying SES. We also know that the patient volume varied largely according to security incidents that made travels difficult no matter which SES a woman had – the one-stop integrated service would therefore make HIV testing more available in contexts where visits to a health facility are potentially risky. This is a strong argument for implementing PITC in free-of-charge sexual and reproductive health structures as a measure to reach women in insecure settings, including women with low SES. PITC would be suitable in a variety of facility types including community-based services as shown in other contexts [32].

The high contraception uptake of over 90% reflects the fact that contraception was the primary reason why patients came. Further, in the current study, particular measures were taken to avoid informal payments for this free service, otherwise common in CAR. Measures were also taken to ensure full confidentiality, which also may have contributed to the high uptake. General health information about HIV and testing was given before enrollment in PITC, which might also have encouraged testing uptake. The study thus shows that HIV testing as part of PITC was effectively implemented in the patient flow. This current study informed practices at the project level and has shaped operations after completion. The data has been used to inform our local partners in CAR including the Ministry of Health. This manuscript aims to share the data and experiences with the greater public health community.

We found a strong and significant association between being married and lower HIV testing uptake. An association between marital status and HIV testing uptake has been found in other studies on VCT [33–35]. It has been explained by married women feeling less at risk of HIV [33], or that partner disclosure may be a barrier for married women to undergo HIV testing [34, 35]. The association between marital status and HIV testing uptake thus necessitates further study. Marital status was also found to be a strong confounder of the association between SES and HIV testing uptake in the current study. This could possibly be explained by the local tradition of dowry, making wealthier men more likely to get married, and married women become part of wealthier families. In poorer families it is common that couples live together and form a family without being legally married. If, as indicated in other studies, married women feel less need to get tested, this may further affect the association between SES and testing uptake.

### Strengths and limitations

The current study is the first to study the association between SES and HIV testing uptake in CAR, and in a conflict setting. It is also one of few studies investigating this association in a clinic-based PITC setting [32, 36, 37]. Further, for the first time a local measure of SES was developed that can be used in future studies. Based on in-depth interviews, this measure was less focused on assets and more focused on social support, access to food and consumption/saving as this was found appropriate in this setting of conflict and looting. All data were collected and encoded specifically for the study, by one single person trained for the study, to reduce information bias. Data collection was done in a confidential room, and the purpose of the study was carefully explained to each participant, in order to avoid stigma around questions revealing poverty and low social status, which could skew data towards a higher SES. Confidentiality of HIV test results was also ensured by having a separate room for the PITC. Questions and translations were thoroughly tested and adapted to ensure understanding and pertinence, and local cultural idiosyncrasies (like calling a partner “husband” in a non-marital relationship) were considered and corrected for during interviews. The participation rate of 98% can be attributed to the fact that MSF is highly trusted after years of providing maternal care in the neighborhood.

There are some limitations. Fifty-nine patients under 18 years were excluded from participation—age in the sample was therefore slightly skewed towards older age. Further, the poorest segment of the population may be under-represented because of opportunity cost of coming to a health facility at all—this group may differ from the sample population regarding testing uptake. As this is a clinic-based sample, we cannot exclude that PITC, on a community level, may be associated with SES in terms of opportunity cost. This should be kept in mind when comparing these findings to cross-sectional studies with random community-based samples.

Religion could be an unmeasured confounder that may influence testing uptake. Religious affiliation was not investigated in this study, because of the sensitivity of religion in this context at the time of the study. There may be an association between religion and marital status in this context, which was shown to be associated with testing uptake. This should be considered in future studies.

In addition to a matrix, PCA was used to find the variables that explained most of the variance in the data. None of the components calculated had high enough eigenvalues. The first component, SES 3, was the closest proxy for SES: six variables explained 16.1% of the variance of the 16 variables used in the analysis. The component did therefore not explain enough variation to be considered reliable. Had we collected a larger number of variables, PCA might have been more suitable. However, minimising time spent on the questionnaire was a priority in this study, to not interfere with the patient flow. Previous MICS studies in CAR have used standardized MICS questionnaires where wealth quintiles are based on the DHS Wealth Index, which uses household assets and utility services as indicator variables [38]. In an unstable setting such as Bangui, this might not fully capture the complex effects of incidents of violence, where physical assets come and go whereas social support, family situation and means of income will be more constant sources of wealth. Including these latter factors can further nuance existing asset-based measures in this context of conflict and violence. As no other studies of SES measures have been performed in CAR, the SES measures developed could be regarded as a starting point to develop asset-based measures with social nuances in further studies.

## Conclusions

This study shows that PITC for HIV can be successfully implemented in the patient flow at a family planning service without affecting family planning uptake, while achieving high HIV testing uptake. In the insecure context of this study, the integration of several services can reduce opportunity cost and risk for the patients and improve HIV testing coverage for women of reproductive age.

Further, no association was found between testing uptake and SES, which shows that when PITC is offered free of charge, without additional wait, low SES is not a barrier to testing uptake. To reach the UNAIDS 90-90-90 goals, we need strategies to increase HIV testing in insecure areas with low testing coverage. Based on this study, we highly recommend this clinic-based PITC setup to reach women of childbearing age in low-income, conflict-afflicted contexts.

### Supplementary Information

**Additional file 1.** Participant characteristics and socio-economic variables (N=1419).

## Data Availability

The anonymous datasets used and analysed during the current study are available from the corresponding author upon reasonable request.

## Annex 1

Patient flow in the clinic.

## Annex 2

Participant form including SES questionnaire. French version.

## Annex 3

SES questionnaire. English version.

## Abbreviations

AIDS: Acquired immunodeficiency syndrome
ART: Antiretroviral treatment
ARV: Antiretroviral
CAR: Central African Republic
CI: Confidence interval
DAG: Directed Acyclic Graph
FCFA: Francs de la Communauté Financière Africaine
HIV: Human immunodeficiency virus
ID: Identification
IDP: Internally displaced population
MICS: Multiple Indicator Cluster Survey
MSF: Médecins Sans Frontières (Doctors Without Borders)
NGO: Non-Governmental Organisation
NSD: Norwegian Centre for Research Data
OR: Odds ratio
PCA: Principal component analysis
PITC: Provider-initiated testing and counselling
PK5: Point Kilométrique 5
PLWH: People living with HIV
REK: Regional Committees for Medical and Health Research Ethics
SDG: Sustainable development goals
SES: Socio-economic status
SD: Standard deviation
SPSS: Statistical package for social sciences
TSD: Tjenester for sensitive data (services for sensitive data)
UN: United Nations
UNAIDS: Joint United Nations Programme on HIV and AIDS
USD: United States Dollars
VCT: Voluntary counselling and testing
WHO: World Health Organisation
