# Supplementary material for: Provider-initiated HIV testing uptake and socio-economic status among women in a conflict zone in the Central African Republic: a mixed-methods cross-sectional study
Source: Confl Health. 2023 Mar 27;17:14. doi: 10.1186/s13031-023-00505-0 (PMC10041765; doi:10.1186/s13031-023-00505-0)
Supplement: Supplementary file 1 — Additional file 1. Participant characteristics and socio-economic variables (N=1419). [file 13031_2023_505_MOESM1_ESM.docx]

| **Variable** | **Category** | **N** | **Percentage** |
| --- | --- | --- | --- |
| Age | 16-20 | 397 | 28.0 |
|  | 21-25 | 552 | 38.9 |
|  | 26-30 | 318 | 22.4 |
|  | 31-35 | 102 | 7.2 |
|  | 36 or above | 50 | 3.5 |
| Education | None | 148 | 10.4 |
|  | Primary school | 446 | 31.4 |
|  | Middle school | 633 | 44.6 |
|  | High school | 172 | 12.1 |
|  | Higher education | 20 | 1.4 |
| Marital status | Single | 339 | 23.9 |
|  | Cohabitating | 783 | 55.2 |
|  | Married | 274 | 19.3 |
|  | Widowed/divorced | 23 | 1.6 |
| Children under 15 | 0 | 75 | 5.3 |
|  | 1 | 395 | 27.8 |
|  | 2 | 378 | 26.6 |
|  | 3 | 281 | 19.8 |
|  | 4 | 182 | 12.8 |
|  | 5 | 71 | 5.0 |
|  | 6 or more | 37 | 2.6 |
| Children to feed | 0 | 1323 | 93.2 |
|  | 1 | 21 | 1.5 |
|  | 2 | 25 | 1.8 |
|  | 3 | 29 | 2.0 |
|  | 4 or more | 21 | 1.5 |
| Source of income | Nothing | 2 | 0.1 |
|  | Agriculture | 17 | 1.2 |
|  | Family member | 671 | 47.3 |
|  | Petty trade or services | 704 | 49.6 |
|  | Paid work | 22 | 1.6 |
|  | Other | 3 | 0.2 |
| Second source of income | Nothing | 760 | 53.6 |
|  | Agriculture | 2 | 0.1 |
|  | Family member | 652 | 45.9 |
|  | Petty trade or services | 4 | 0.3 |
|  | Paid work | 0 | 0 |
|  | Other | 1 | 0.1 |
| Saving per week | None | 1008 | 71.0 |
|  | Up to 1000 FCFA | 36 | 2.5 |
|  | 1000-2900 FCFA | 196 | 13.8 |
|  | 3000-5900 FCFA | 104 | 7.3 |
|  | Over 5900 FCFA | 75 | 5.3 |
| Phone use | No phone | 770 | 54.3 |
|  | 0 FCFA | 31 | 2.2 |
|  | 1-499 FCFA | 150 | 10.6 |
|  | 500-999 FCFA | 287 | 20.2 |
|  | 1000-1499 FCFA | 92 | 6.5 |
|  | 1500-1999 FCFA | 49 | 3.5 |
|  | 2000 FCFA or more | 40 | 2.8 |
| Head of household | Participant | 85 | 6.0 |
|  | Husband | 265 | 18.7 |
|  | Partner | 652 | 45.9 |
|  | Parents | 313 | 22.1 |
|  | Grandparents | 34 | 2.4 |
|  | Other | 70 | 4.9 |
| % of household members  under 15 | 0-33.3 | 173 | 12.2 |
|  | 33.4-44.4 | 384 | 27.1 |
|  | 44.5-50 | 338 | 23.8 |
|  | 50.1-60 | 277 | 19.5 |
|  | Over 60 | 247 | 17.4 |
| Household size | 1-3 | 161 | 11.3 |
|  | 4-7 | 688 | 48.5 |
|  | 8-12 | 360 | 25.4 |
|  | 13 or more | 210 | 14.8 |
| Household members  per breadwinner | 1-2.3 | 76 | 5.4 |
|  | 2.4-5.7 | 793 | 55.9 |
|  | 5.8-9 | 400 | 28.2 |
|  | 10 or more | 150 | 10.6 |
| Type of housing | Owned house | 663 | 46.7 |
|  | Rented house | 739 | 52.1 |
|  | Host family | 16 | 1.1 |
| Time since last move | Don’t know | 6 | 0.4 |
|  | Less than 2 months | 39 | 2.7 |
|  | 2-6 months | 153 | 10.8 |
|  | 7-12 months | 174 | 12.3 |
|  | 13 months – 5 years | 645 | 45.5 |
|  | 6-10 years | 142 | 10.0 |
|  | 11 years or more | 239 | 16.8 |
| Address | 3e arrondissement | 539 | 38.0 |
|  | 5e arrondissement | 234 | 16.5 |
|  | 6e arrondissement | 147 | 10.4 |
|  | Bimbo | 413 | 29.1 |
|  | Other | 86 | 6.1 |
| Days per week with enough food | None | 0 | 0.0 |
|  | 1-3 days | 467 | 32.9 |
|  | 4-6 days | 94 | 6.6 |
|  | Every day | 858 | 60.5 |
| Last food shortage | Yesterday | 84 | 5.9 |
|  | This week | 300 | 21.2 |
|  | Last week | 178 | 12.5 |
|  | Last month or longer | 855 | 60.3 |
